# Supplementary material for: Spatio-temporal modelling for the evaluation of an altered Indian saline Ramsar site and its drivers for ecosystem management and restoration
Source: PLoS One. 2021 Jul 22;16(7):e0248543. doi: 10.1371/journal.pone.0248543 (PMC8297798; doi:10.1371/journal.pone.0248543)
Supplement: S1 Protocol — (PDF) [file pone.0248543.s001.pdf]

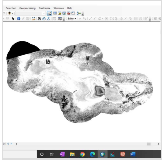

Jun 12, 2021

Spatio-temporal modelling for future prediction of wetlands

Rajashree Naik[1], Laxmi Kant Sharma[1]

[1]Central University of Rajasthan

1 Works for me Share

dx.doi.org/10.17504/protocols.io.bvryn57w

Central University of Rajasthan

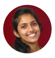

Rajashree Naik
Central University of Rajasthan

DISCLAIMER

Use the licensed version of software.

ABSTRACT

This protocol is intended to help the wetland researchers willing to use remote sensing data. It is designed for conducting past, present and future geospatial modelling. It involves step by step methods for collection of satellite data, processing of data, conducting research, accuracy assessment, future prediction and map composition using Arc GIS, ERDAS Imagine and Terrset software. The expected outcomes will be the Land Use Land Cover maps of past, present and future at spatio-temporal scale in .tif, .img or .png format. Area of each class, rate of change, area transition matrix and the wetland change driving factors will also be obtained. The only limitation is to have prior field knowledge for the wetland for better results.

ATTACHMENTS

protocol_LULC.docx

DOI

dx.doi.org/10.17504/protocols.io.bvryn57w

PROTOCOL CITATION

Rajashree Naik, Laxmi Kant Sharma 2021. Spatio-temporal modelling for future prediction of wetlands. **protocols.io**
https://dx.doi.org/10.17504/protocols.io.bvryn57w

MANUSCRIPT CITATION please remember to cite the following publication along with this protocol

Naik R, SHARMA L. Spatio-temporal modelling for the evaluation of an altered Indian saline Ramsar site and its drivers for ecosystem management and restoration. bioRxiv. 2021 Jan 1.

LICENSE

This is an open access protocol distributed under the terms of the Creative Commons Attribution License, which permits unrestricted use, distribution, and reproduction in any medium, provided the original author and source are credited

CREATED

Jun 12, 2021

LAST MODIFIED

Jun 12, 2021

Citation: Rajashree Naik, Laxmi Kant Sharma (06/12/2021). Spatio-temporal modelling for future prediction of wetlands. https://dx.doi.org/10.17504/protocols.io.bvryn57w

This is an open access protocol distributed under the terms of the **Creative Commons Attribution License** (https://creativecommons.org/licenses/by/4.0/), which permits unrestricted use, distribution, and reproduction in any medium, provided the original author and source are credited

PROTOCOL INTEGER ID

50712

GUIDELINES

1. Visit the wetland prior to starting the research.
2. Collect field photographs and GPS location of different Land Use Land Cover classes.
3. Use half of the GPS as training datasets and half for accuracy assessment.
4. Maintain the uniformity of datasets for future prediction in TerrSet software

MATERIALS TEXT

1. Geocoordinates of study area
2. Data: Landsat (MSS, TM, ETM+ and OLI)
3. Software: Arc GIS, ERDAS Imagine, TerrSet

Arc GIS 10.5
Windows 10
by ESRI

ERDAS Imagine 2014
Windows 10
by ERDAS, Inc.

TerrSet 2020
Windows 10
by Clark Labs

1. Toposheets
2. GPS
3. MS Office

Microsoft 2013 and above
Windows 10
by Microsoft

SAFETY WARNINGS

Always keep a backup of all the input datasets and output maps.

DISCLAIMER:

Use the licensed version of software.

Citation: Rajashree Naik, Laxmi Kant Sharma (06/12/2021). Spatio-temporal modelling for future prediction of wetlands.
https://dx.doi.org/10.17504/protocols.io.bvryn57w

This is an open access protocol distributed under the terms of the **Creative Commons Attribution License** (https://creativecommons.org/licenses/by/4.0/), which permits unrestricted use, distribution, and reproduction in any medium, provided the original author and source are credited

BEFORE STARTING

1. Install licensed version software like Arc GIS, ERDAS Imagine and TerrSet.
2. Learn to operate GPS before going to field.

Study area

1

1. Decide the wetland to be studied.
2. Collect Toposheet.
3. Georeference and mosaic in Arc GIS.
4. Digitize the official boundary of the wetland.
5. Shape file is ready in .shp format (vector layer).

Data collection

2

1. Create an account and login to [USGS Earth Explorer]
2. Enter the name of the study area, and thedata range or the time period of study in the space provided.
3. 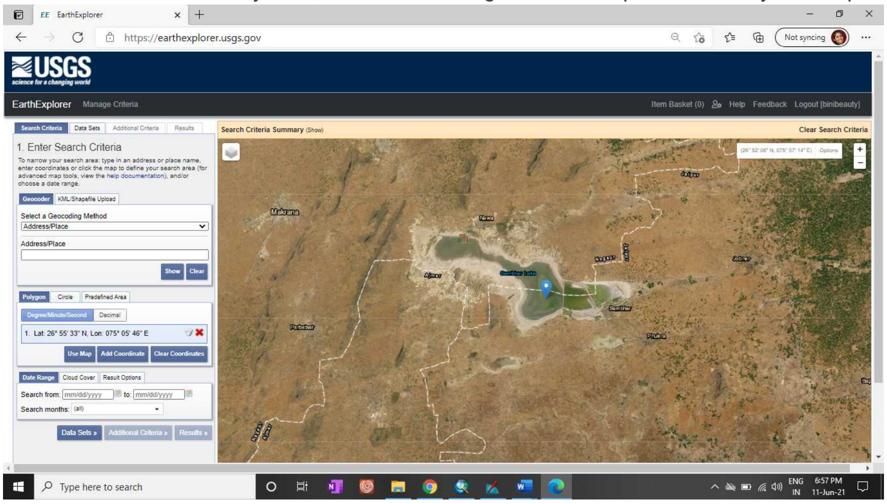

USGS Earth Explorer interface

4. In the second tab named “Dataset”, select “Declassified” for the aerial photograph “CORONA” and “Landsat” for satellite images.
5. Select “**Landsat Collection 2 Level 1**” for collecting Multispectral Scanner (MSS) data and “Landsat Collection 2 Level 2” for Thematic Mapper (TM), Enhanced Thematic Mapper Plus (ETM+) and Operational Land Imager (OLI) datasets.
6. Then click on the “Result” option given below to visualize the results.

Download the tiles after checking the metadata

Data processing

3

1. Unzip the downloaded tiles.
2. “**Layer stack**” the respective band numbers representing Blue, Green, Red and NIR bands.
3. Create True Color Composite (TCC) and False Color Composite (FCC) for visualization and LULC class identification.
4. “**Subset**” the study area using the prepared boundary shapefile.

Citation: Rajashree Naik, Laxmi Kant Sharma (06/12/2021). Spatio-temporal modelling for future prediction of wetlands.
https://dx.doi.org/10.17504/protocols.io.bvryn57w

This is an open access protocol distributed under the terms of the **Creative Commons Attribution License** (https://creativecommons.org/licenses/by/4.0/), which permits unrestricted use, distribution, and reproduction in any medium, provided the original author and source are credited

5. 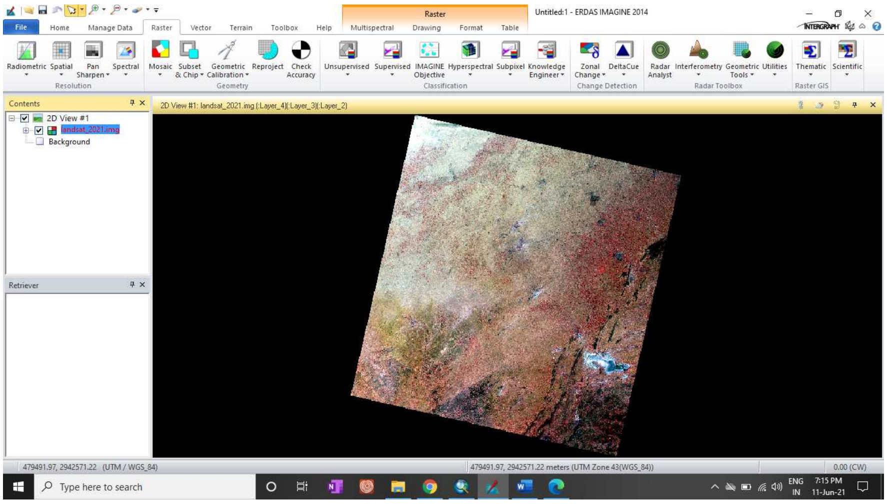

Interface of ERDAS Imagine with FCC of Sambhar Lake

LULC classification

4
1. Select “**Raster tab**”, then “**Supervised menu**” and then “**Signature editor**”.
2. Select minimum 20 to 30 signature for each LULC classes using the training datasets obtained from field.
3. Save the Signature file.
4. Select “Supervised Classification”.
5. Import subset image of study area as input file and also import signature file.
6. Set Parametric rule as “Maximum Likelihood”.
7. Give a name for the output file.
8. Run the program.
9. Obtain the classified map and do accuracy assessment.
10. Calculate the total area of each class.
11. Repeat the steps for all the desired years of study.
12. 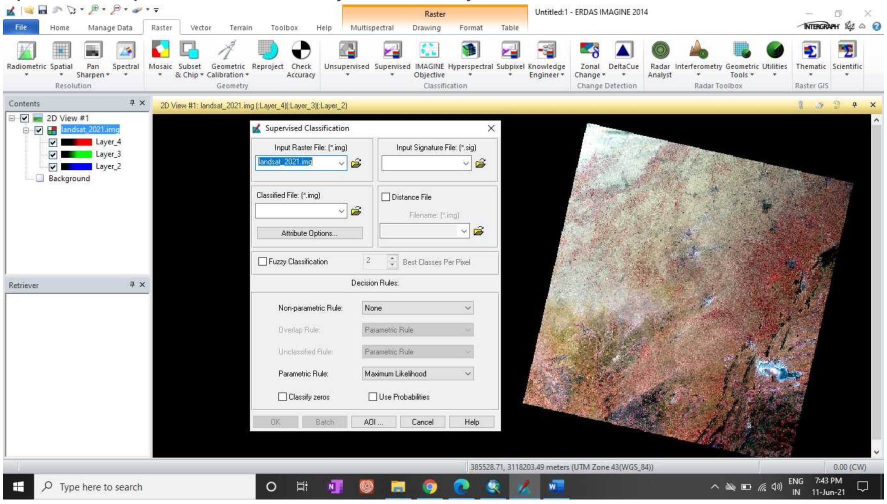

Window for supervised classification.

13.

Change detection

5
1. Select the “**Raster Tab**”.
2. Select “**Thematic**” menu.

Citation: Rajashree Naik, Laxmi Kant Sharma (06/12/2021). Spatio-temporal modelling for future prediction of wetlands. https://dx.doi.org/10.17504/protocols.io.bvryn57w

This is an open access protocol distributed under the terms of the **Creative Commons Attribution License** (https://creativecommons.org/licenses/by/4.0/), which permits unrestricted use, distribution, and reproduction in any medium, provided the original author and source are credited

3. Select “**Matrix Union**”.
4. Import two classified images of different years.
5. Give output file name.
6. Select “Union” in Area type and “Unsigned 16 bit” in data type.
7. Run the program
8. Export transition matrix output file to MS Excel sheet
9. Calculate rate of change

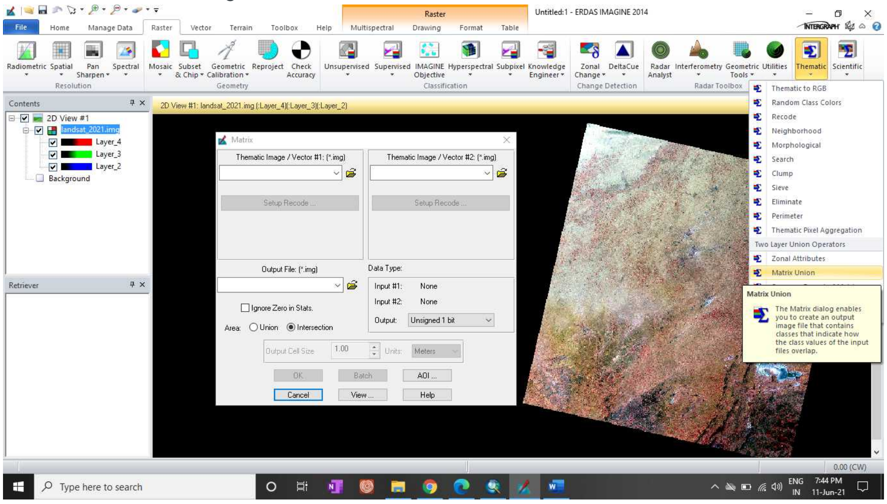

Window for change detection in ERDAS Imagine

Future prediction

6

1. Open TerrsSet Geospatial Monitoring and Modeling software.
2. Import two classified maps in .tif format.
3. Open "**Land Change Modeler**".
4. Create new session and name it.
5. Obtain “**Change maps**”, “**Change analysis**” and “**Spatial trend of change**”.

Citation: Rajashree Naik, Laxmi Kant Sharma (06/12/2021). Spatio-temporal modelling for future prediction of wetlands. https://dx.doi.org/10.17504/protocols.io.bvryn57w

This is an open access protocol distributed under the terms of the **Creative Commons Attribution License** (https://creativecommons.org/licenses/by/4.0/), which permits unrestricted use, distribution, and reproduction in any medium, provided the original author and source are credited

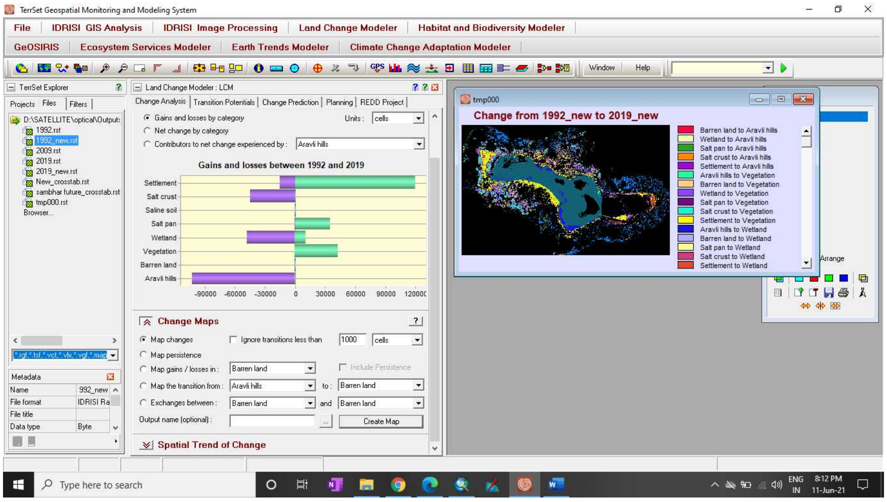

Future prediction results in TerrSet software

CORONA Aerial Photograph

7
1. Add downloaded Declassified CORONA photograph.
2. Georeference it.
3. To the required form using "**Clip**" option of Raster in Data Management tools.
4. Digitize and classify using visual interpretation keys.
5. Calculate area using scale "Calculate geometry" in the attribute table.
6. Compose final map.

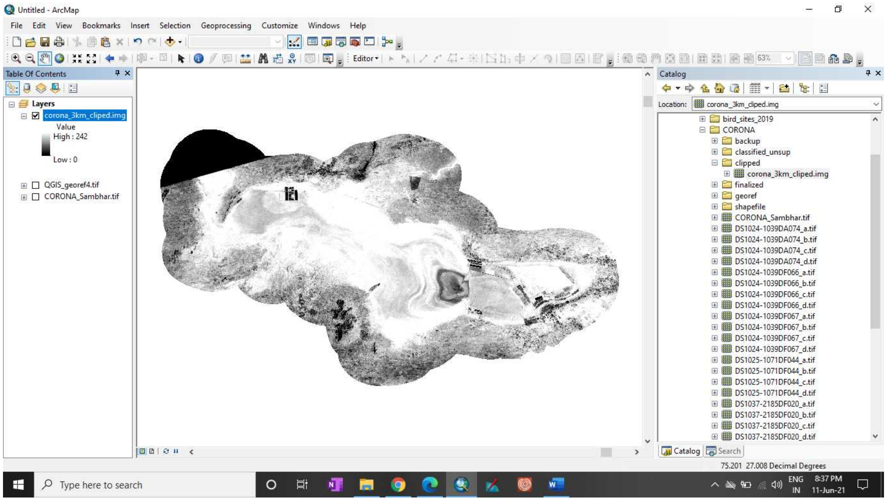

Declassified CORONA photograph

Map composition

8
1. Add the Maps to Arc GIS.
2. Switch over to "**Layout View**".
3. Go to "**Insert**" menu.

Citation: Rajashree Naik, Laxmi Kant Sharma (06/12/2021). Spatio-temporal modelling for future prediction of wetlands. https://dx.doi.org/10.17504/protocols.io.bvryn57w

This is an open access protocol distributed under the terms of the **Creative Commons Attribution License** (https://creativecommons.org/licenses/by/4.0/), which permits unrestricted use, distribution, and reproduction in any medium, provided the original author and source are credited

4. Insert Map elements like Legends, North Arrow, Scale bar and text, Grid.
5. "**Export**" the final composed map in tiff format.

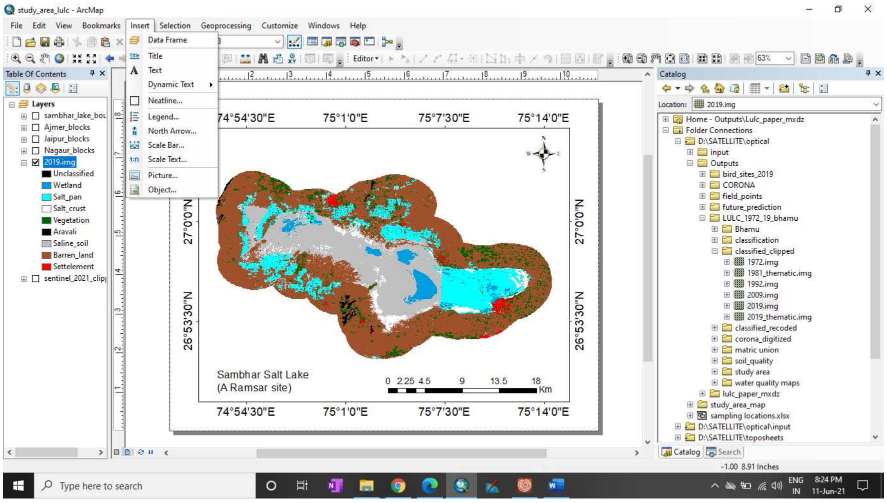

Elements of finally composed map in Arc GIS

Soil, water and bird study

9
1. Soil and water sample were analyzed using the protocols of APHA [1].
2. Bird census were conducted using protocol of [2].

10

Citation: Rajashree Naik, Laxmi Kant Sharma (06/12/2021). Spatio-temporal modelling for future prediction of wetlands.
https://dx.doi.org/10.17504/protocols.io.bvryn57w

This is an open access protocol distributed under the terms of the **Creative Commons Attribution License** (https://creativecommons.org/licenses/by/4.0/), which permits unrestricted use, distribution, and reproduction in any medium, provided the original author and source are credited
