# Supplementary material for: Spatio-temporal modelling for the evaluation of an altered Indian saline Ramsar site and its drivers for ecosystem management and restoration
Source: PLoS One. 2021 Jul 22;16(7):e0248543. doi: 10.1371/journal.pone.0248543 (PMC8297798; doi:10.1371/journal.pone.0248543)
Supplement: S2 Protocol — (DOCX) [file pone.0248543.s002.docx]

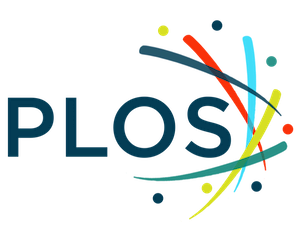


Lab Protocol Article Template

Title Spatio-temporal modelling for future prediction evaluation wetlands

Metadata

Funding No funding has been received.

Competing interests: The authors certify that there is no competing interest to disclose.

Data availability

Data is provided as supplementary file with all the raw and processed datasets. File named as S1 datasets.

Associated content

dx.doi.org/10.17504/protocols.io.bvryn57w

Abstract

This protocol is intended to help the wetland researchers willing to use remote sensing data. It is designed for conducting past, present and future geospatial modelling. It involves step by step methods for collection of satellite data, processing of data, conducting research, accuracy assessment, future prediction and map composition using Arc GIS, ERDAS Imagine and Terrset softwares. The expected outcomes will be the Land Use Land Cover maps of past, present and future at spatio-temporal scale in .tif, .img or .png format. Area of each class, rate of change, area transition matrix and the wetland change driving factors will also be obtained. The only limitation is to have prior field knowledge for the wetland for better results.

Introduction

Traditionally, wetland studies were conducted using expensive, tedious and time-consuming field methods. However, with the widespread use of remote sensing and Geographic Information Technology (GIS), landscape level studies at different spatio-temporal scales are also possible. This protocol was developed for conducting research on India’s largest inland saline wetland, Sambhar Salt Lake from 1963 to 2059 at decadal scale. It differs from the other protocols as it involves integration of satellite datasets with aerial photograph of CORONA of 1963, before the start of any satellite mission by 1972. The only limitation of the protocol is that the study was conducted only during winter season when migratory birds visited the lake, unlike summer when the lake is completely dry.

Materials and Methods

Section 1: Study area

1. Decide the wetland to be studied.
2. Collect Toposheet.
3. Georeference and mosaic in Arc GIS.
4. Digitize the official boundary of the wetland.
5. Shape file is ready in .shp format (vector layer).

Section 2: Data collection

1. Create account and login to <https://earthexplorer.usgs.gov/>
2. Enter the name of study area, and data range or the time period of study in the space provided.
3. In the second tab named “Dataset”, select “Declassified” for aerial photograph “CORONA” and “Landsat” for satellite images.
4. Select “Landsat Collection 2 Level 1” for collecting Multispectral Scanner (MSS) data and “Landsat Collection 2 Level 2” for Thematic Mapper (TM), Enhanced Thematic Mapper Plus (ETM+) and Operational Land Imager (OLI) datasets.
5. Then click on the “Result” option given below to visualize the results.
6.
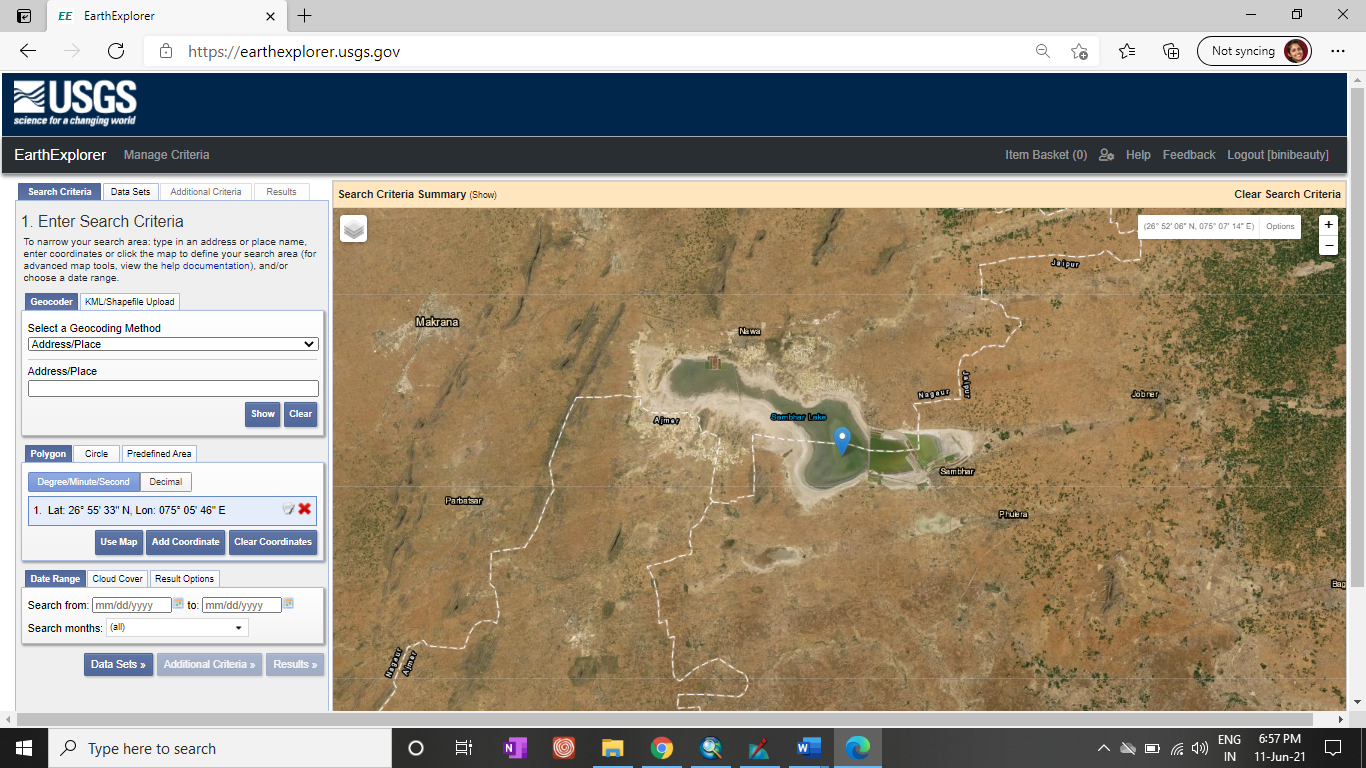
Download the tiles after checking the metadata.

**Fig 1 USGS Earth Explorer interface.**

Section 3: Data processing (ERDAS Imagine 2014)

1. Unzip the downloaded tiles.
2. “Layer stack” the respective band numbers representing Blue, Green, Red and NIR bands.
3. Create True Color Composite (TCC) and False Color Composite (FCC) for visualization and LULC class identification.
4.
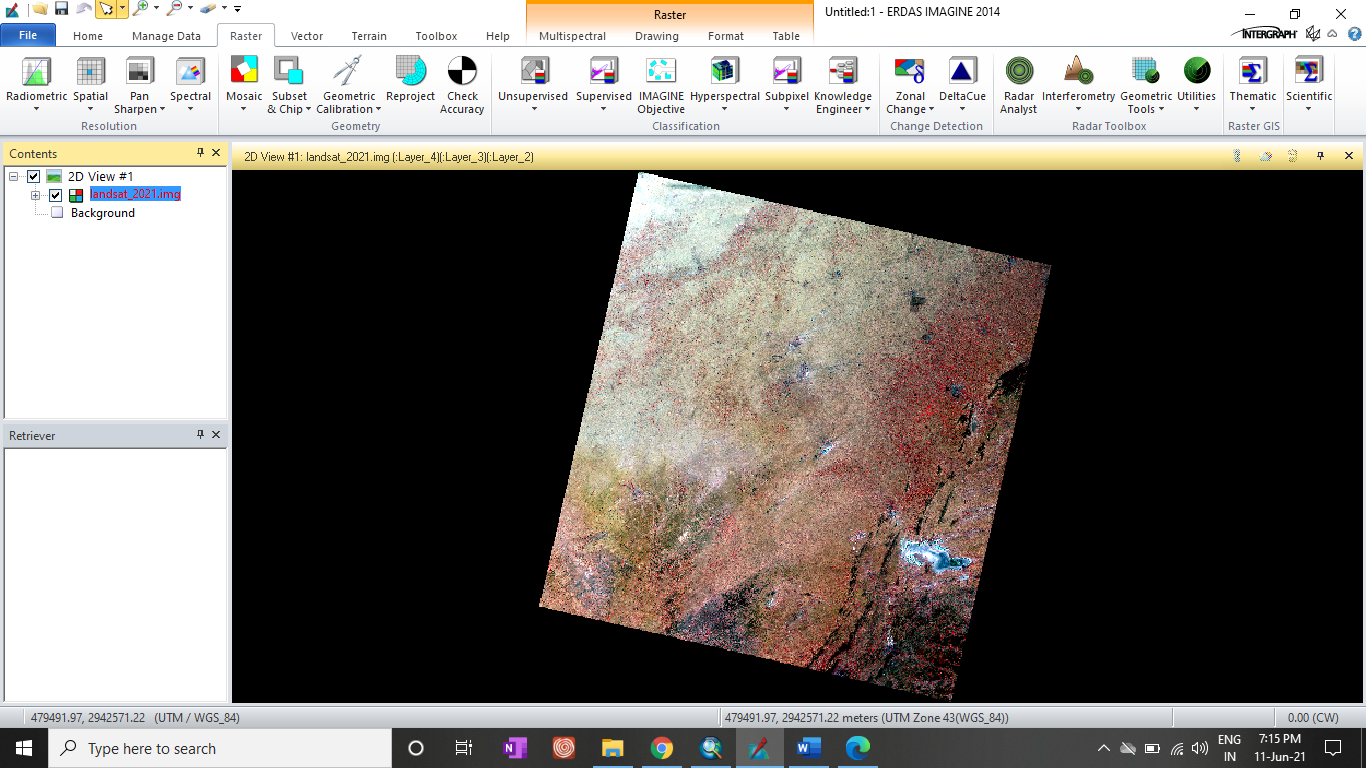
“Subset” the study area using the prepared boundary shapefile.

**Fig 2 Interface of ERDAS Imagine with FCC of Sambhar Lake.**

Section 4: Classification (Supervised)

1. Select “Raster tab”, then “Supervised menu” and then “Signature editor”.
2. Select minimum 20 to 30 signature for each LULC classes using the training datasets obtained from field.
3. Save the Signature file.
4. Select “Supervised Classification”.
5. Import subset image of study area as input file and also import signature file.
6. Set Parametric rule as “Maximum Likelihood”.
7. Give name for output file.
8. Run the program.
9. Obtain the classified map.
10. Calculate the total area of each class.
11.
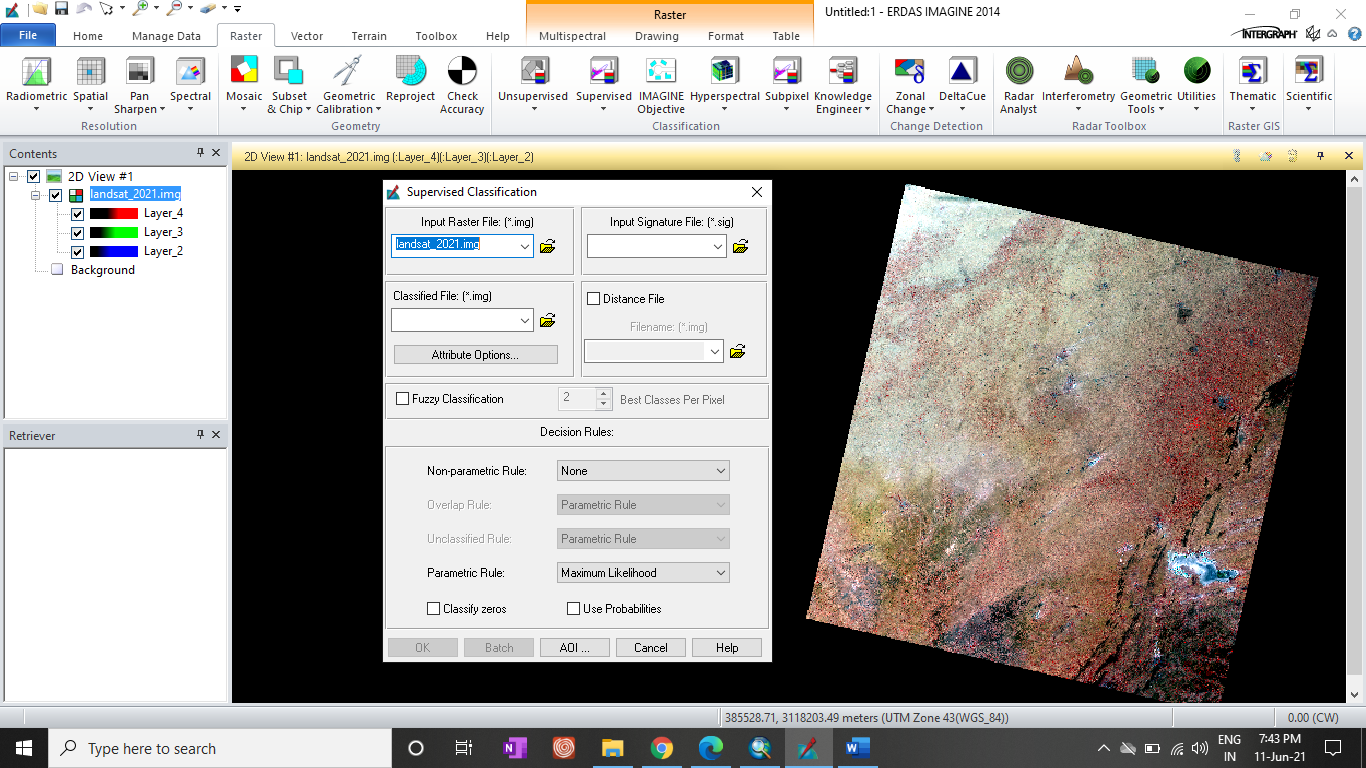
Repeat the steps for all the desired years of study.

**Fig 3 Window for supervised classification.**

Section 5: Change detection

1. Select the “Raster Tab”.
2. Select “Thematic” menu.
3. Select “Matrix Union”.
4. Import two classified images of different years.
5. Give output file name.
6. Select “Union” in Area type and “Unsigned 16 bit” in data type.
7. Run the program.
8. Export the transition matrix output file into MS Excel.
9. Calculate rate of change in MS Excel.

**
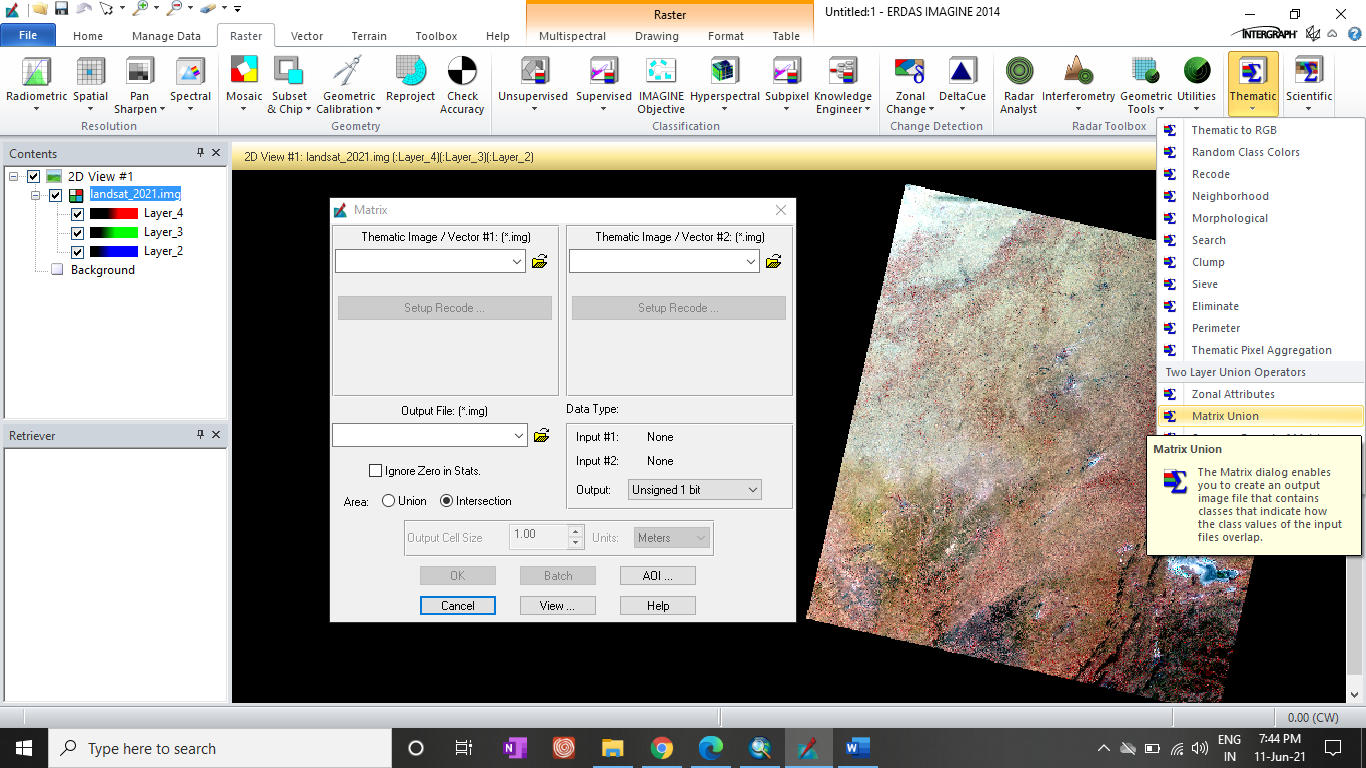
Fig 4 Window for change detection in ERDAS Imagine.**

Section 6: Future prediction

1. Open TerrSet Geospatial Monitoring and Modeling software.
2. Import two classified maps in .tif format.
3. Open the “Land Change Modeler”.
4. Create New session and name it.
5. Import “Earlier” and “Later” LULC classified images and continue.
6.
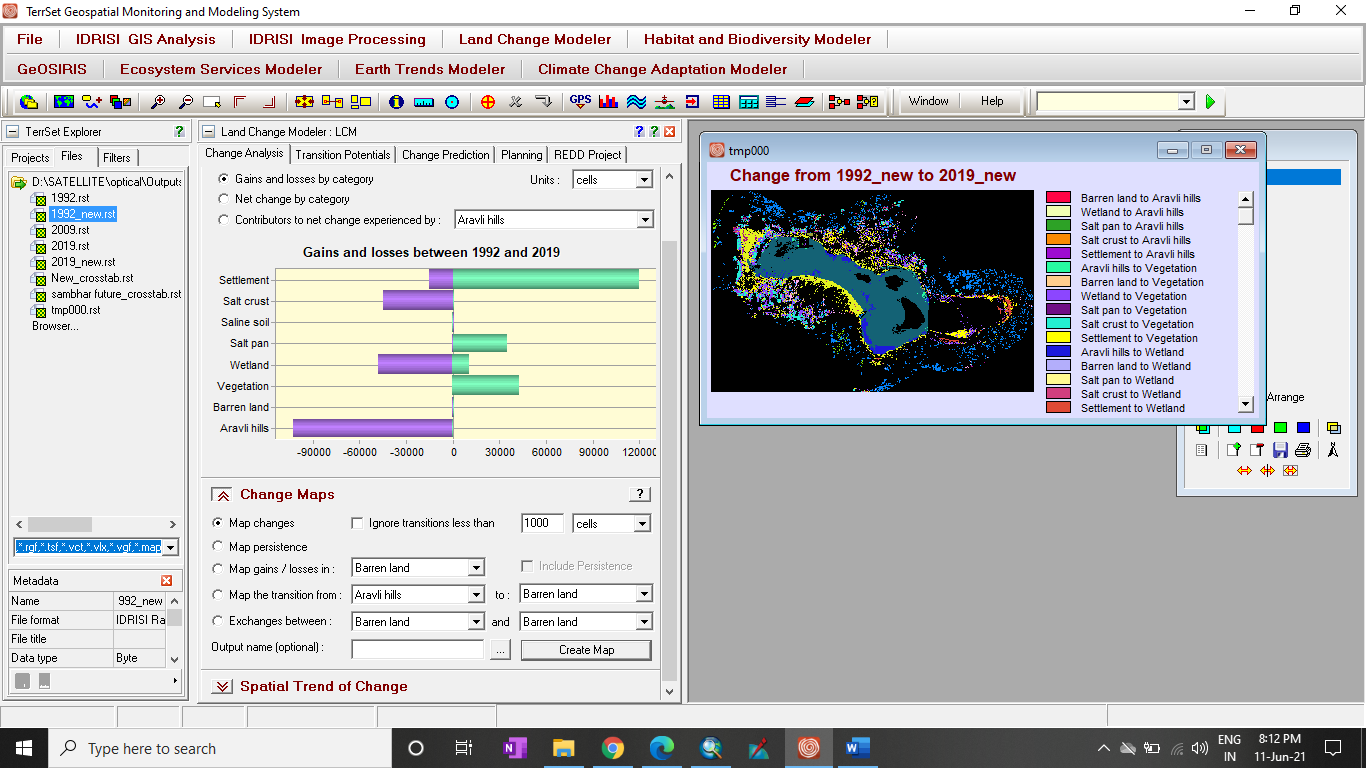
Obtain “Change maps”, “Change analysis” and “Spatial trend of change”.

**Fig 5 Future prediction results in TerrSet software.**

Section 7: CORONA aerial photograph

1. Add the downloaded CORONA declassified image.
2. Georeference it.
3. To the required form using “Clip” option of Raster in Data Management tools.
4. Digitize and classify using visual interpretation keys.
5. Calculate area using scale “Calculate geometry” in the attribute table.
6.
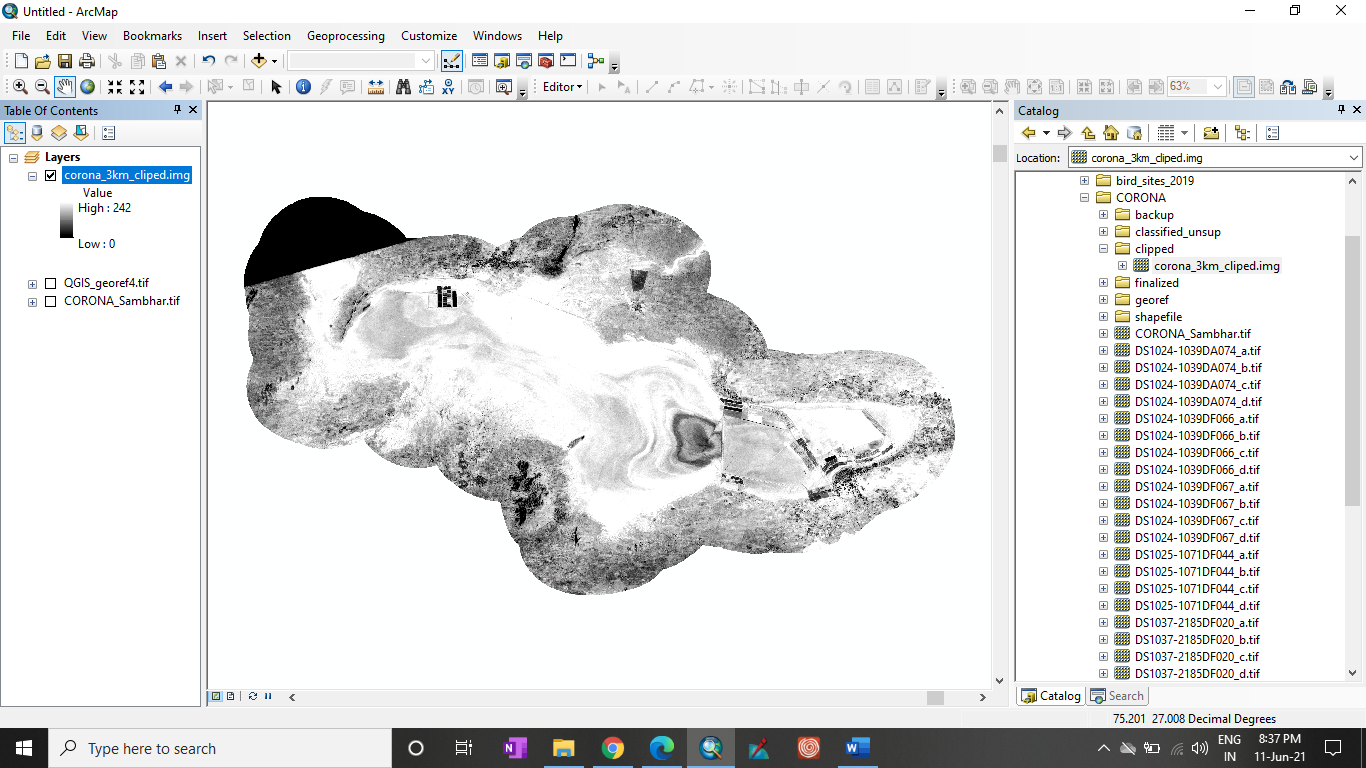
Compose final map.

**Fig 6 Declassified CORONA photograph.**

Section 8: Map Composition (Arc GIS)

1. Add the Maps to Arc GIS.
2. Switch over to “Layout View”.
3. Go to “Insert” menu. Insert Map elements like Legends, North Arrow, Scale bar and text, Grid.
4. “Export” the final composed map in tiff format.

**
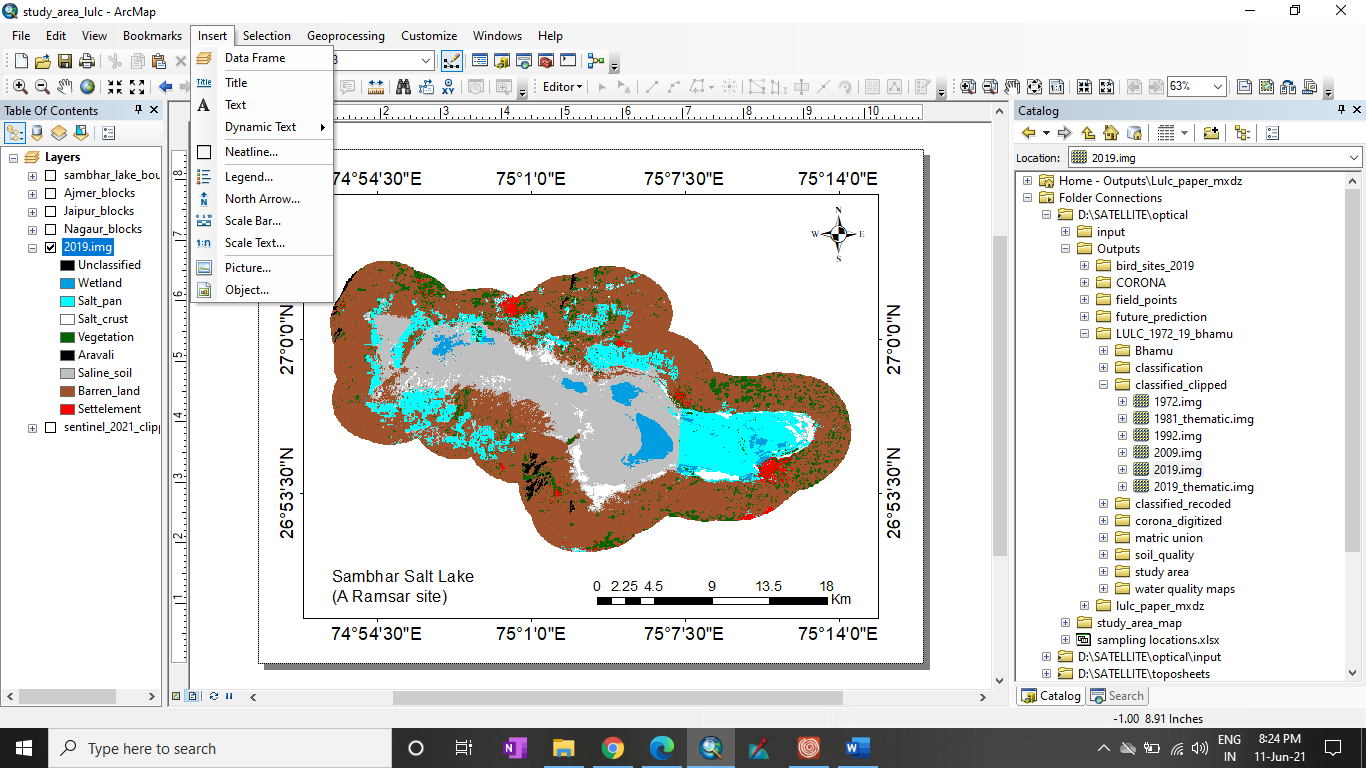
Fig 7 Elements of finally composed map in Arc GIS.**

Section 9: Soil, Water and Bird study

1. Soil and water sample were analyzed using the protocols of APHA [1].
2. Bird census were conducted using protocol of [2].

Expected results

The expected results are

1. Classified maps of past time period.
2. Change maps for compared time periods.
3. Future prediction maps.
4. Area of each class in each time period.
5. Transition matrix of area.
6. Rate of change.

Ethics declarations

Use of licensed version of softwares are to be used.

Supporting information

S1: Step-by-step protocol.

Acknowledgements

We acknowledge Remote Sensing and GIS laboratory of Department of Environmental Science, School of Earth Sciences, Central University of Rajasthan for providing all the required hardware and software for conducting this protocol.

Authors’ contributions

Both the authors contributed to the study conception, design, and data collection. Protocol was conceptualized by Sharma LK and was finalized and performed by Naik R.

References

Baird RB. Standard methods for the examination of water and wastewater, 23rd. Water Environment Federation, American Public Health Association, American Water Works Association; 2017. <https://doi.org/https://doi.org/10.2105/SMWW.2882.216>

Bibby CJ, Burgess ND, Hill DA, Hillis DM, Mustoe S. Bird census techniques. Elsevier; 2000 Aug 29.
