# Supplementary material for: Spatio-temporal modelling for the evaluation of an altered Indian saline Ramsar site and its drivers for ecosystem management and restoration
Source: PLoS One. 2021 Jul 22;16(7):e0248543. doi: 10.1371/journal.pone.0248543 (PMC8297798; doi:10.1371/journal.pone.0248543)
Supplement: Attachment — Submitted filename: reviewer FIGURE 1.pdf [file pone.0248543.s005.pdf]

Figure 1

Click here to access/download;Figure;Fig 1 study area.tif

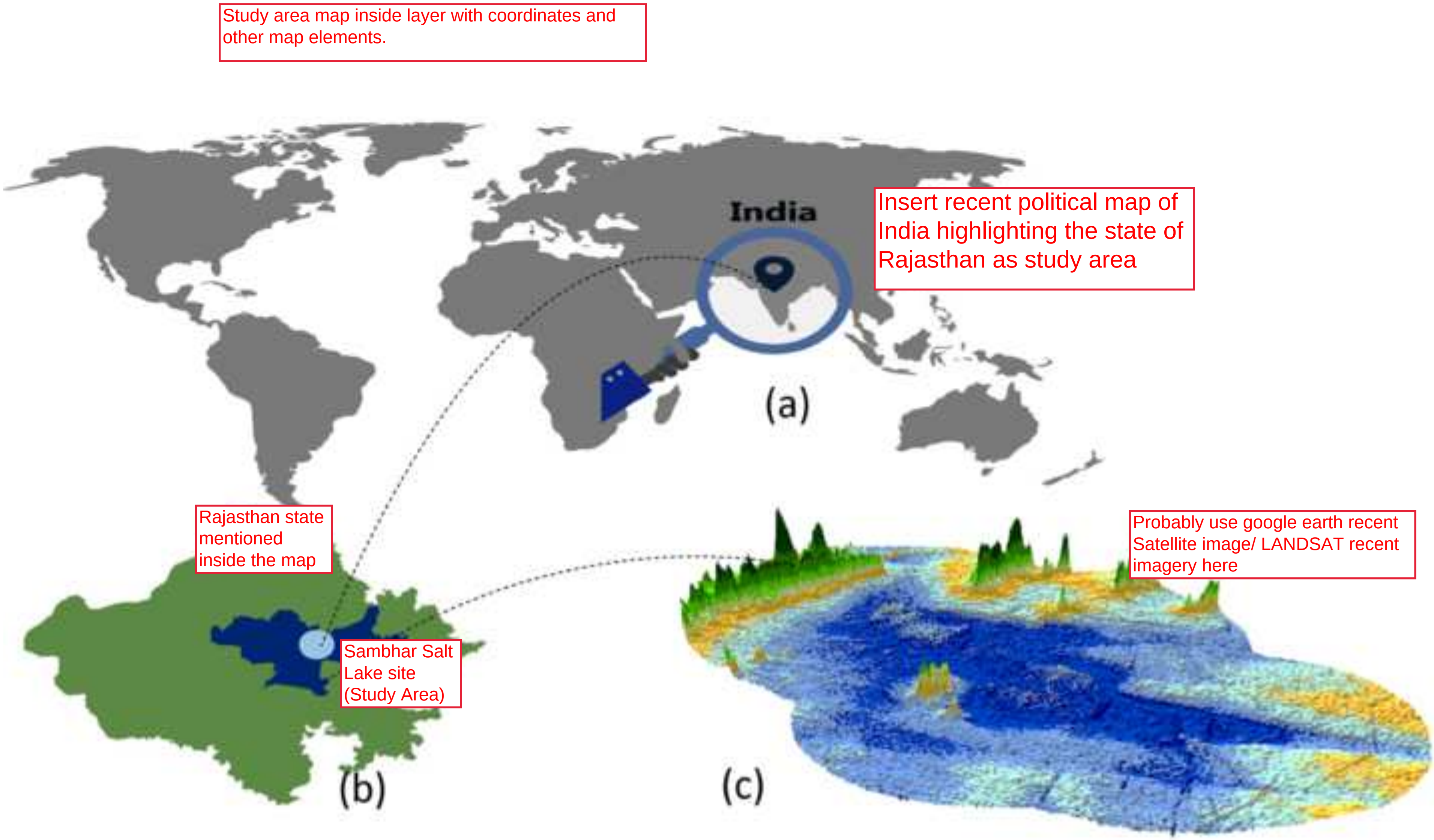

Figure 2

Click here to access/download;Figure;Fig 2 Methodology.tif

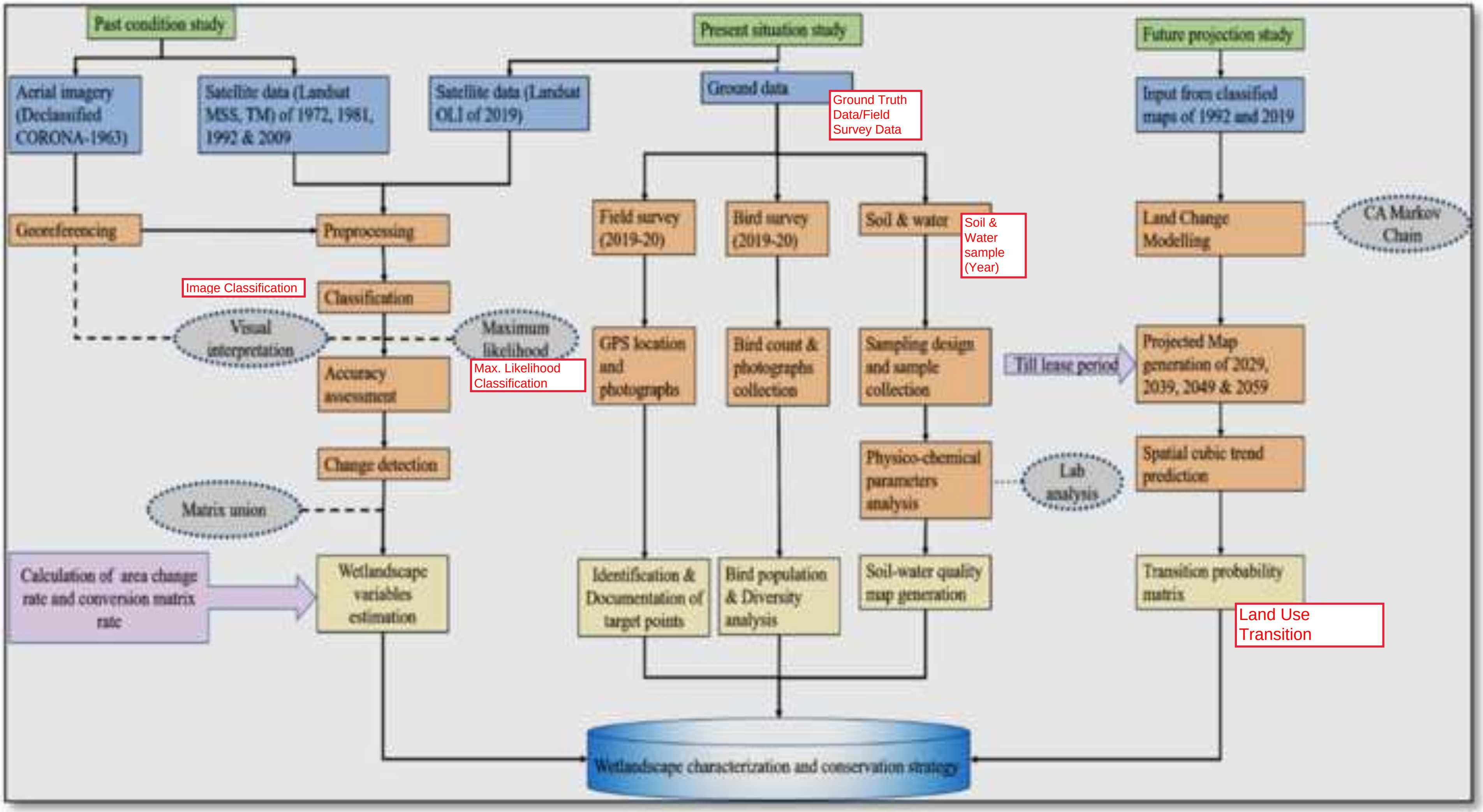

Figure 3

Click here to access/download;Figure;Fig 3 field locations.tif

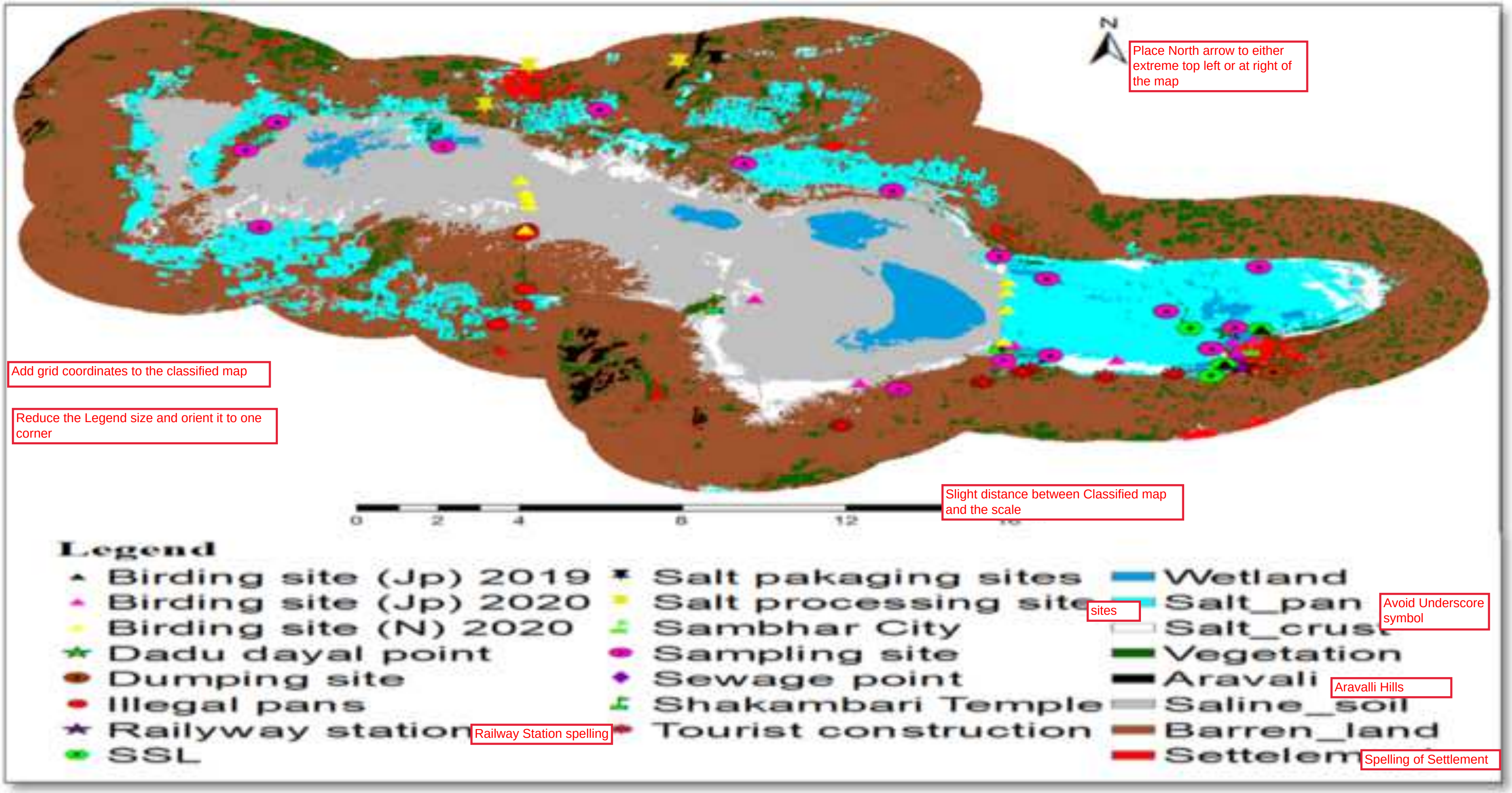

Figure 4 Click here to access/download;Figure;Fig 4 field photographs.tif

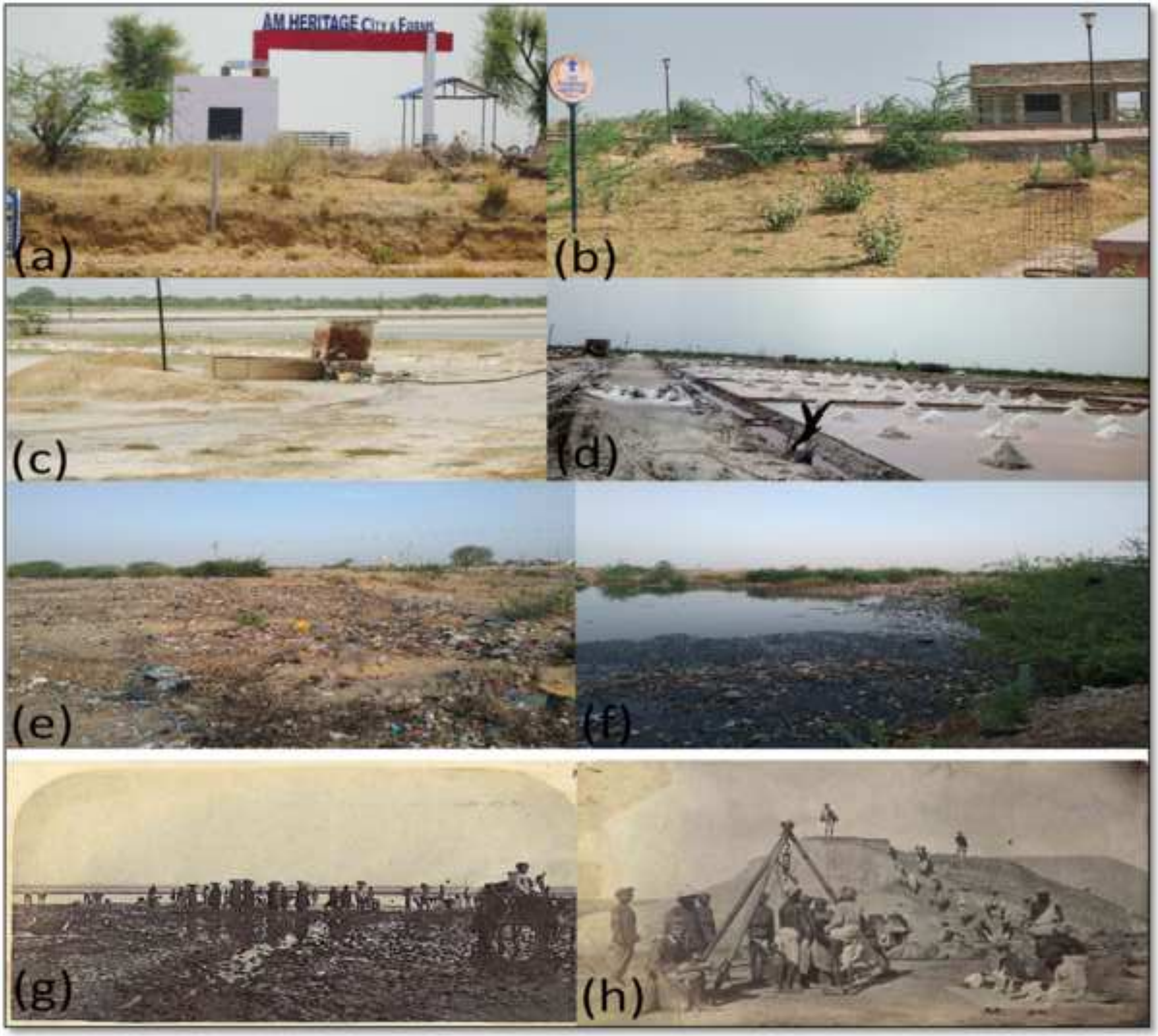

Figure 5

Click here to access/download;Figure;Fig 5 past lulc.tif

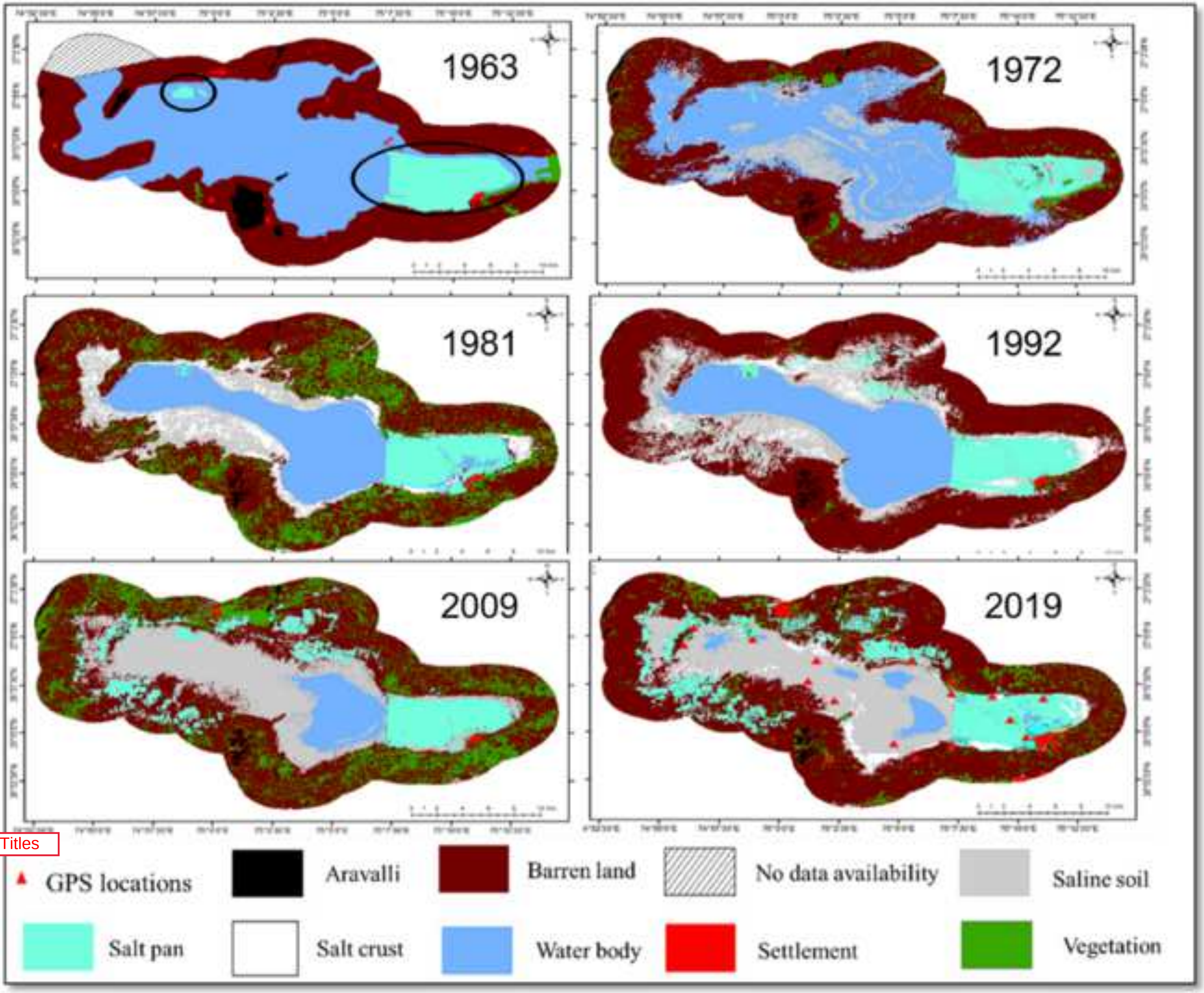

Figure 6

Click here to access/download;Figure;Fig 6 trend graph.tif

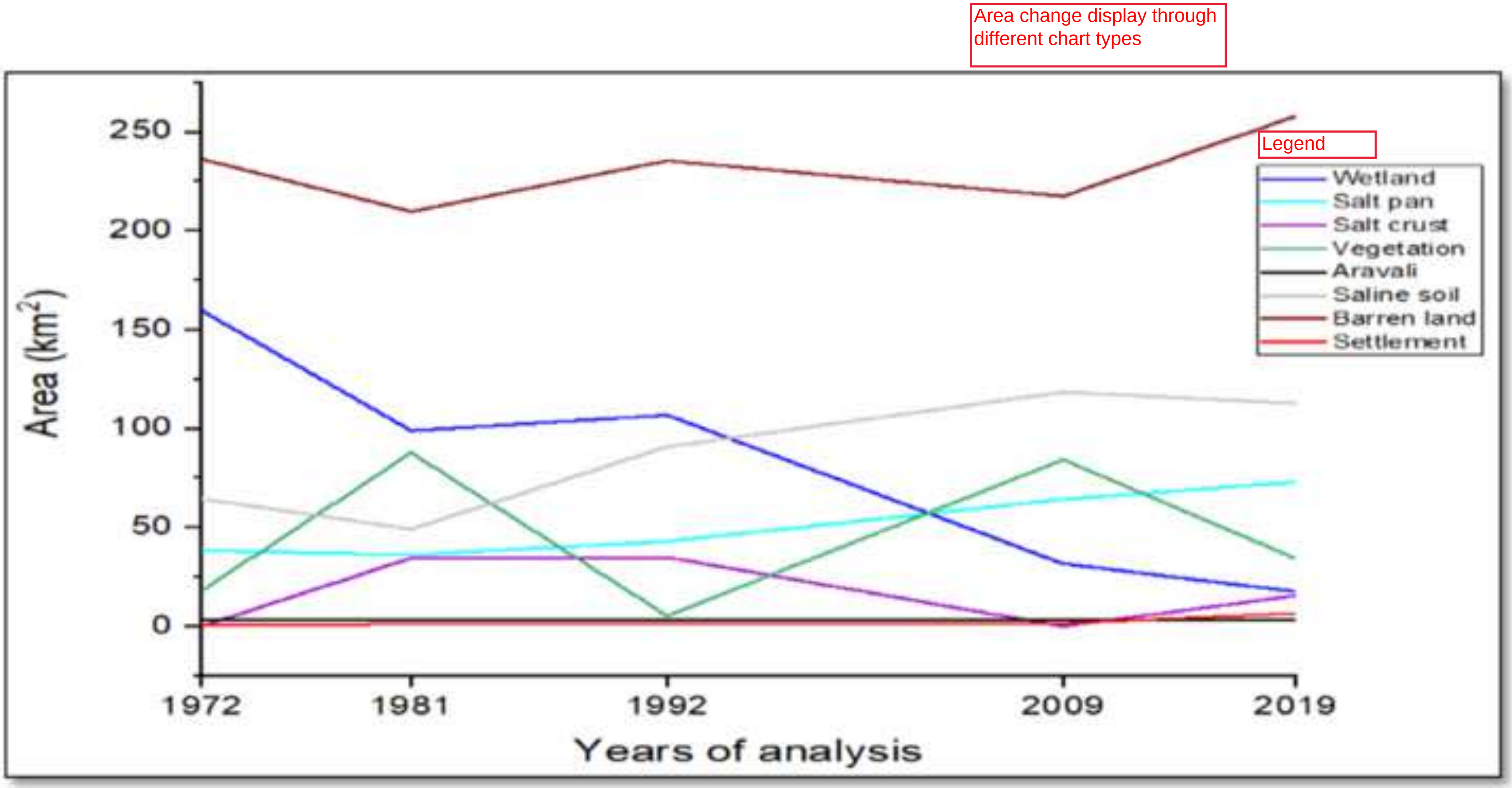

Figure

Click here to access/download;Figure;Fig 7 soil maps.tif

Insert multiple map layers with their respective grids, north arrow and legends for the respective maps.

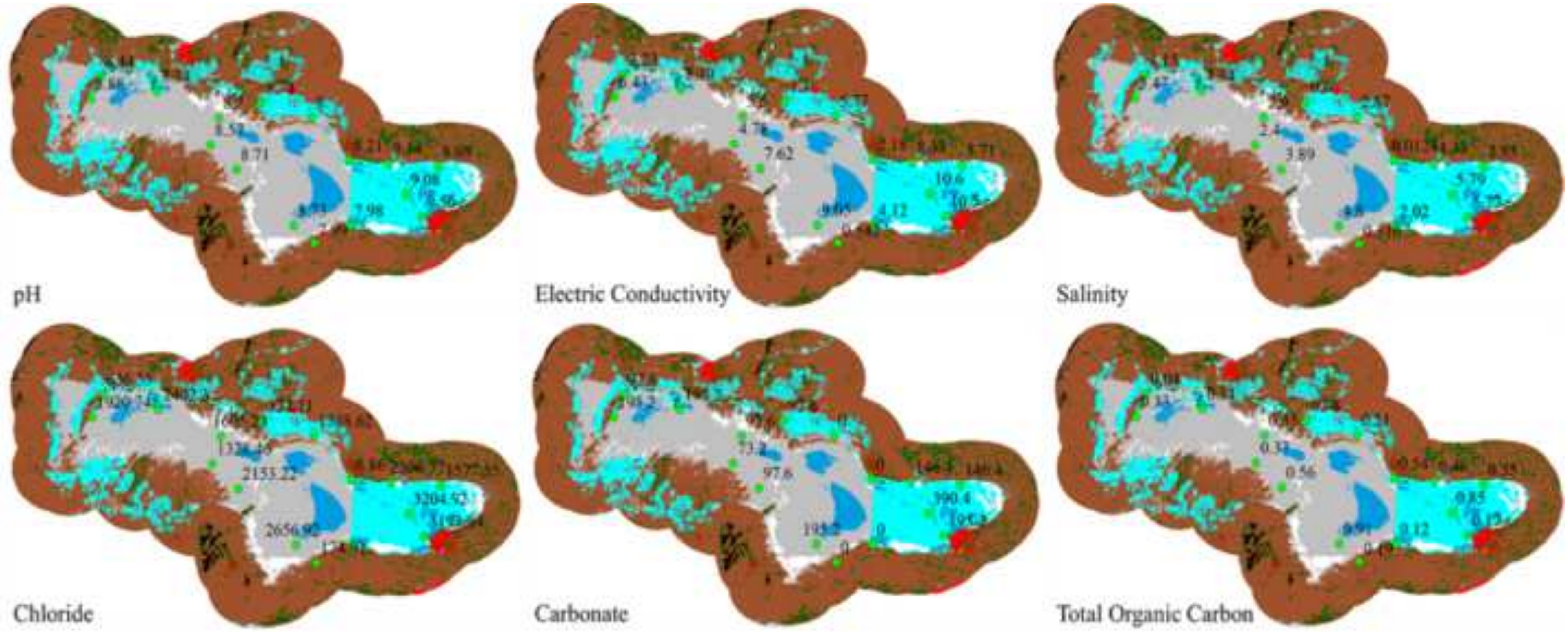

Figure 8

Click here to access/download;Figure;Fig 8 water maps.tif

Insert map layers and its elements for the water parameters

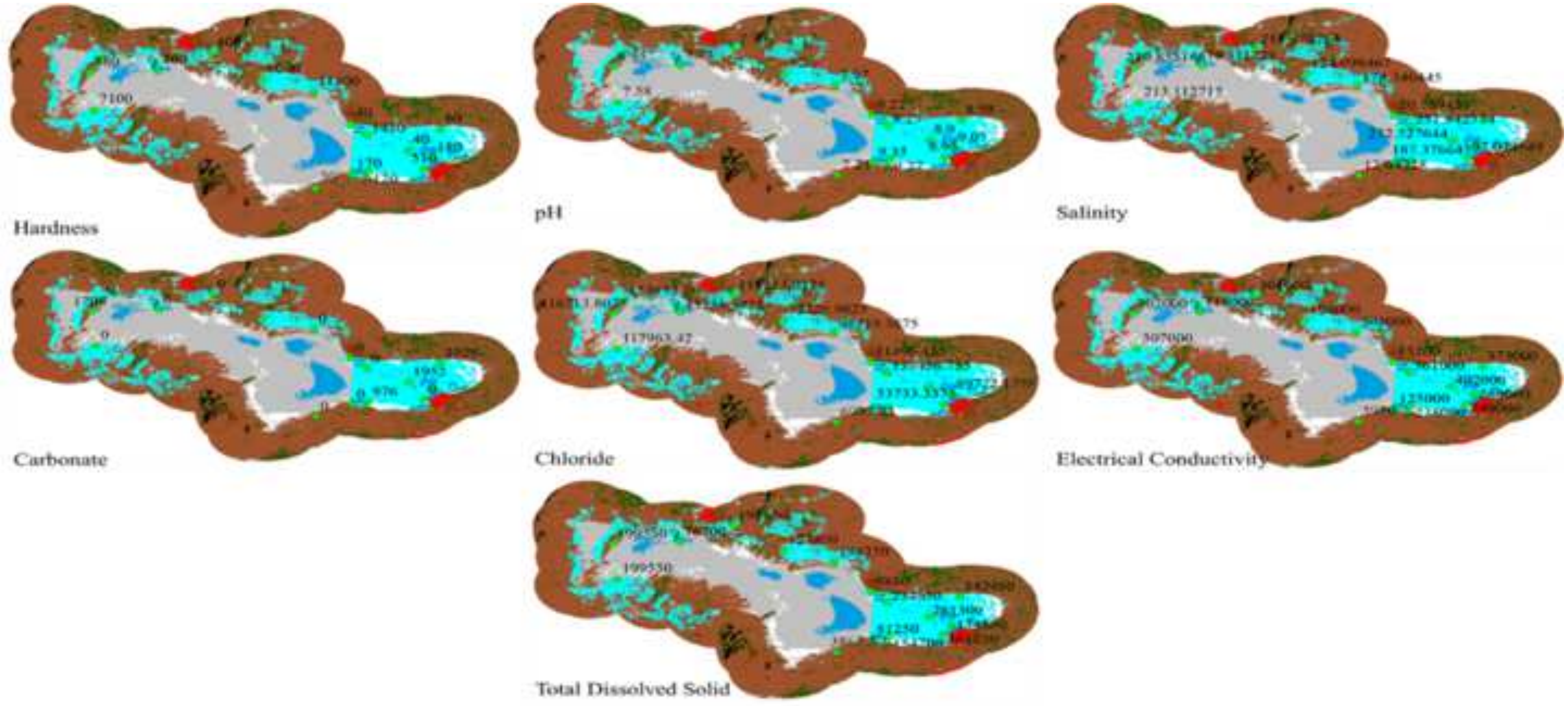

Click here to access/download;Figure;Fig 9 future maps.tif

Insert grid, north arrow. scale and legends for respective Future projection maps with individual year wise title.

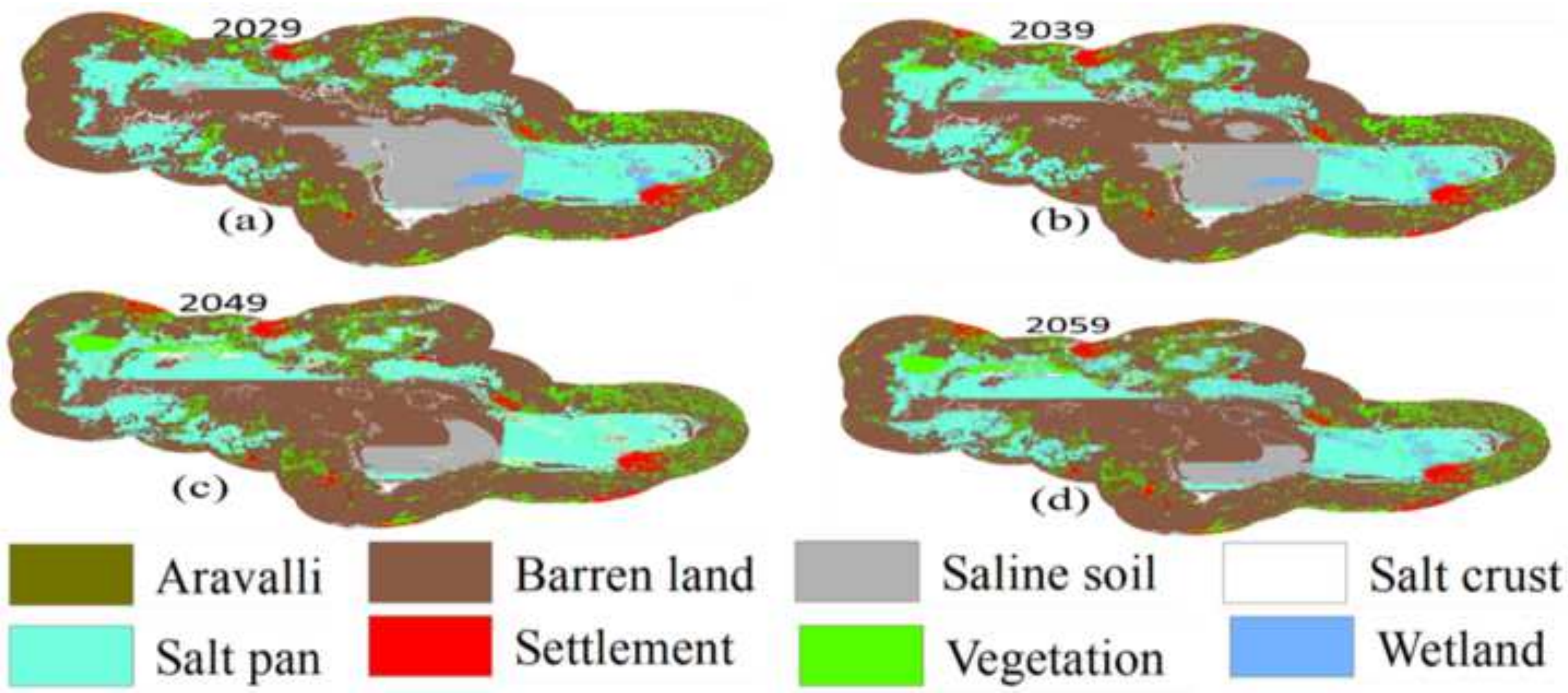

Figure

Click here to access/download;Figure;Fig 10 gain offset graphs.tif

Figure numbering to be done below the gain offset graph

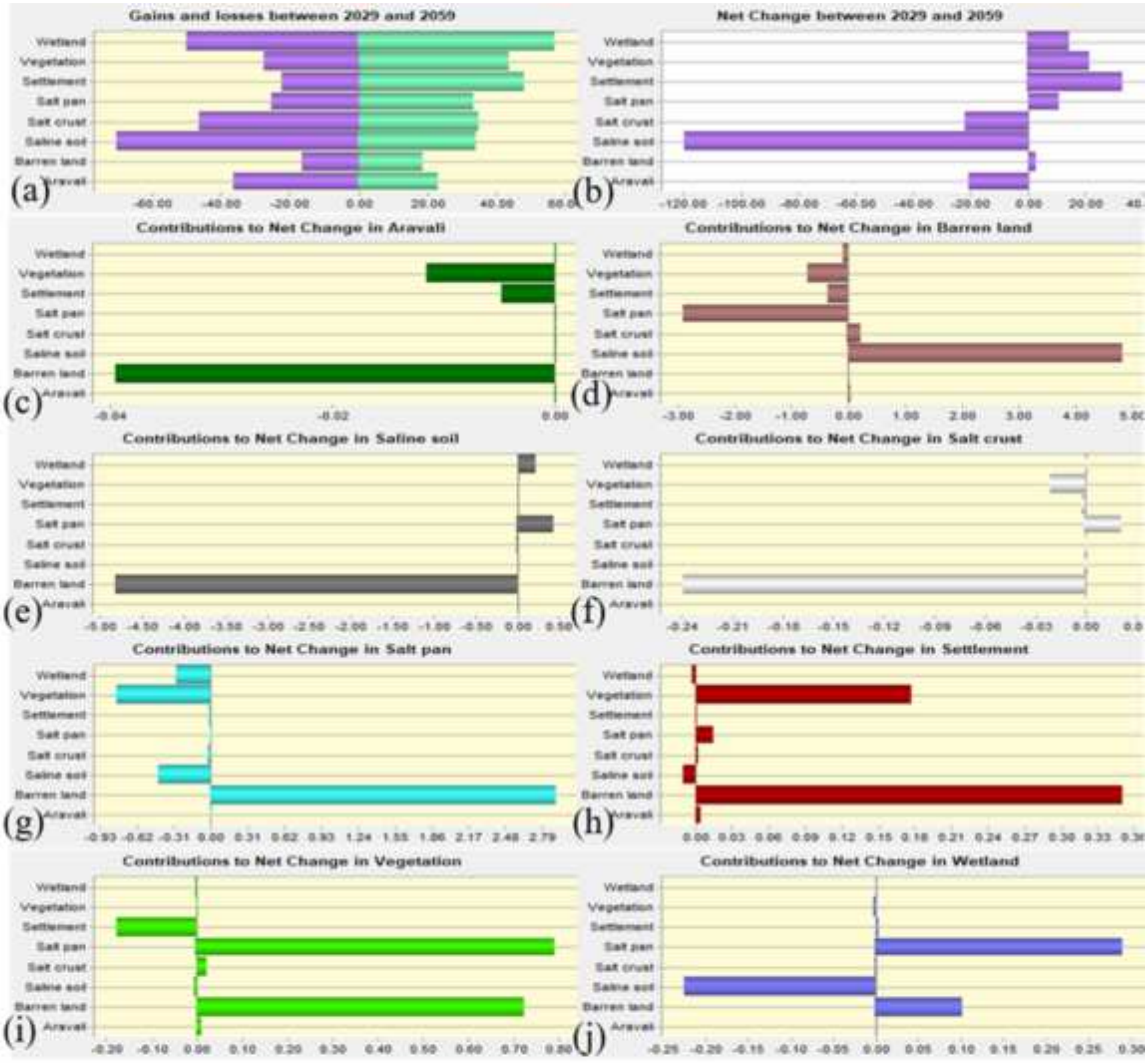

Figure

Click here to access/download;Figure;Fig 11 spatial cubic maps.tif

mention year on the cubic trend (From-Till)

title of the analysis below to be;
Cubic Trend: wetland to Salt Pan
Cubic Trend: Wetland to Settlement
Cubic Trend: Wetland to Barren Land and so on.........

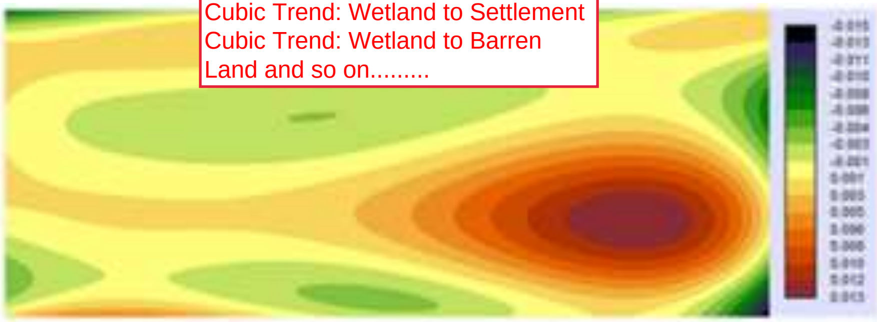

Cubic trend for conversion of wetland to salt pan

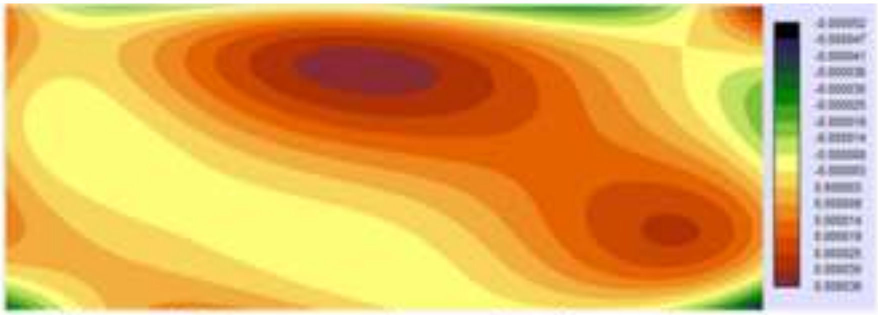

Cubic trend for conversion wetland to settlement

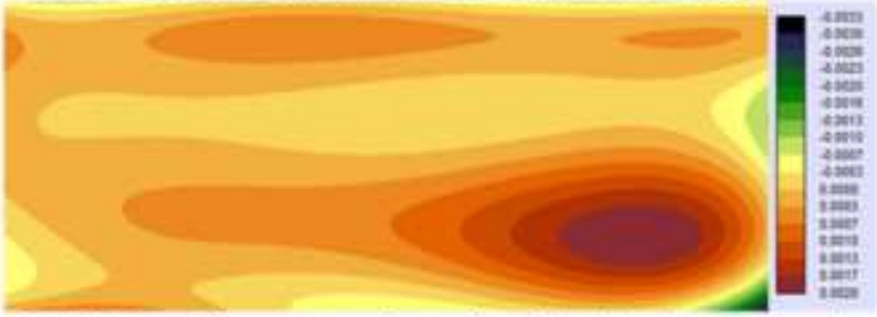

Cubic trend for conversion of wetland to barren land

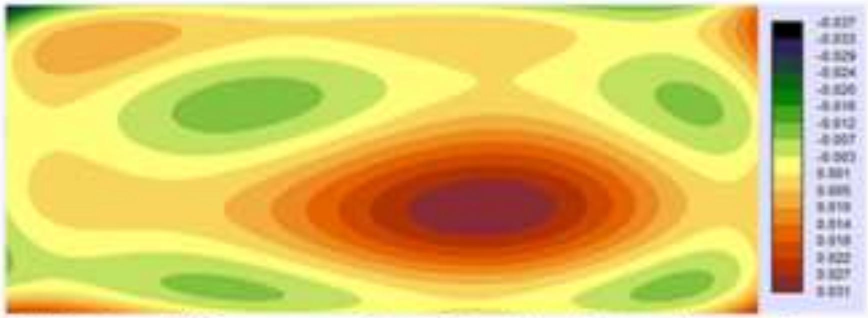

Cubic trend for conversion of wetland to saline soil

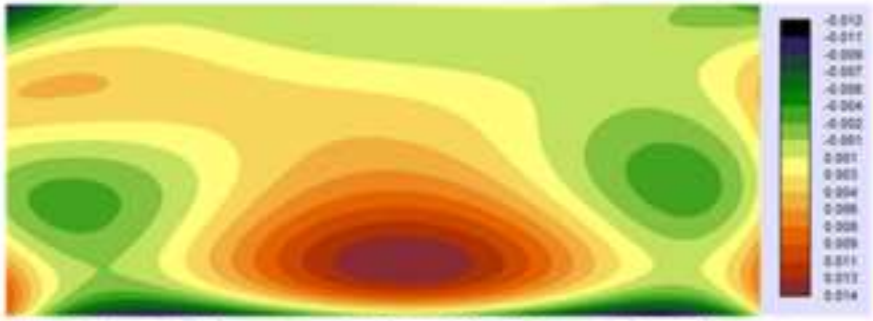

Cubic trend for conversion of salt crust to salt pan

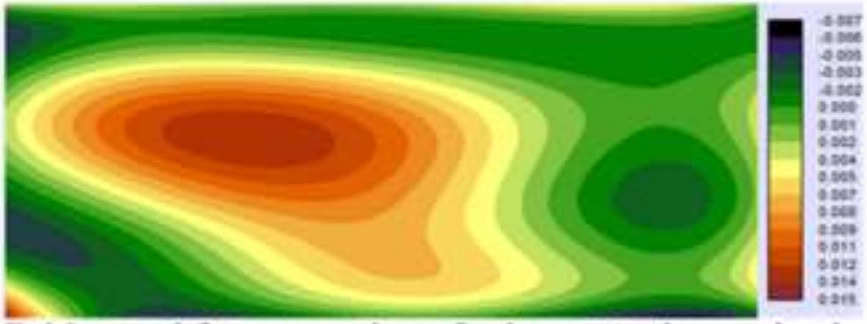

Cubic trend for conversion of salt crust to barren land

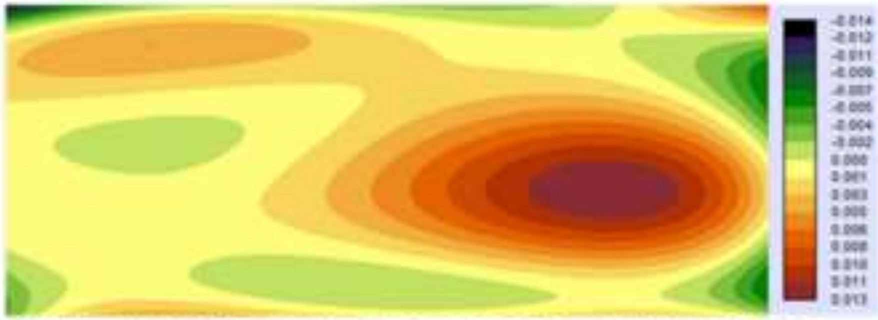

Cubic trend for conversion of saline soil to salt pan

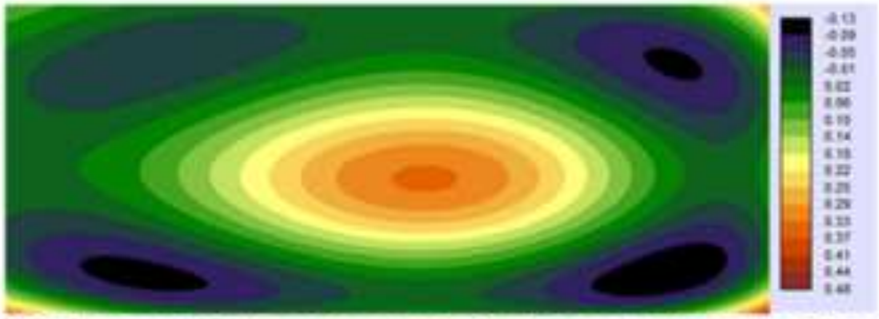

Cubic trend for conversion of saline soil to barren land
