# Supplementary material for: Spatio-temporal modelling for the evaluation of an altered Indian saline Ramsar site and its drivers for ecosystem management and restoration
Source: PLoS One. 2021 Jul 22;16(7):e0248543. doi: 10.1371/journal.pone.0248543 (PMC8297798; doi:10.1371/journal.pone.0248543)
Supplement: Attachment — Submitted filename: 2_Response to reviewers.docx [file pone.0248543.s006.docx]

**Date:** 16 June 2021

**Title:** Spatio-temporal modelling for the evaluation of an altered Indian saline Ramsar site and its drivers for ecosystem management and restoration

**Manuscript Number**: PONE-D-21-05894

**Subject**: Response to suggestions

Dear Reviewers,

We deeply express our gratitude towards your continuous and tiring efforts to read, understand, analyze our manuscript with id “PONE-D-21-05894” and provide suggestion. Your noble suggestions have tremendously improved the quality of the work. The suggestion for graphics included modification of study area map, Land Use Land Cover maps, soil and water quality maps, field visit maps, research methodology flowchart and graphs which were highly creditable. Further, suggestion for writeup included modification of abstract, introduction, correction of figure numbering, experimental testbeds, and processing techniques for satellite images.

All the suggestions have been accepted and incorporated to the best of our knowledge. We have segregated the comments according to the reviewers and provided the details of each action taken, and their location in the manuscript in a tabular form provided in the next section.

Once again, we humbly thank you for the strenuous efforts you have taken and for the patience to receive our response.

|  | **Reviewer #1** | | | |
| --- | --- | --- | --- | --- |
|  | **Comment** | | | **Response** |
|  | The manuscript titled " Spatio-temporal modelling for the evaluation of an altered Indian saline Ramsar site and its drivers for ecosystem management and restoration"  I feel that the paper has the merit and nicely written. The comments have been raised in the attached MS itself. Figures require a lot of changes and additions for clarity.  Once taken care of, the paper may be considered for publication. | | | Suggestions incorporated in the Figures, graphs and maps |
| **S.No.** | **Comments** | **Reply to each Comment** | **Action taken** | **Line no. the revised Manuscript** |
| 1 | 1963 or 1969 CORONA aerial image | Answered | It is 1963 | 18 |
| 2 | Study area map on recent political map of India highlighting state of Rajasthan as study area | Accepted | Prepared new study area map | 135-137 |
| 3 | Map title to be written inside the map of Rajasthan state and study area (Ramsar sites) | Accepted | Incorporated | 135-137 |
| 4 | Area calculation through scale factor | Accepted | Calculated | 151-153 |
| 5 | Insert multiple map layers with their grids, north arrow and legends for the respective maps. | Accepted | Incorporated | - |
| 6 | Figure 9 or 10, not in sync with the Figures pdf | Accepted | Rectified | 310 |
|  | **Reviewer #2** | | |  |
| **S.No.** | **Comments** | **Reply to each Comment** | **Action taken** | **Line no. the revised Manuscript** |
| 1 | Abstract need to be highlighted with the achieved experimental outcome. | Accepted and re-written | More clear sentences are added and explained | 13-32 |
| 2 | In the Introduction section, authors need to high   a) "What is the main contribution of the manuscript", b) "What problem is identified from the related work used in the manuscript" and c) "Explained the structures of manuscript" | Accepted and answered | Introduction section is completely modified and required answers are written | 36-101 |
| 3 | There are introductory lines between the section and sub-section | Accepted and removed | Accepted and removed | - |
| 4 | a) Figure 2, Methodology, what technique is used to process the data?   and   b) What problems identified by the authors can be solved by data preprocessing techniques? | a) Accepted and incorporated b) Accepted and mentioned | b) A new line on necessity of pre-processing is added | a) 163-166  b) mentioned in Methodology figure 2 |
| 5 | All figures used in this manuscript are very low quality, it need to replace with the 300 dpi images. | Accepted and incorporated | - | - |
| 6 | To analysis the results, authors used the kappa coefficient. There are more statistical parameters, authors should also consider that parameters. | Accepted with regret | Due to COVID-19 pandemic lockdown, University lab is closed. It is not possible for the authors to repeat the software-based work currently at home. | - |
| 7 | a) What is the evaluation testbed for the experimental analysis? b) Which kind of software is used to achieve that output? c) It is more beneficial for the other researchers. | Answered | a) For the soil-water parameters accepted method of APHA was followed,  b) For remote sensing and GIS, ARC GIS, ERDAS Imagine, and TerrSet were used  c) Yes, all the methods can be replicated by any researchers for any lake ecosystems of the world | - |
| 8 | Conclusion section need to be partially modified with experimental outcome from the study? | Accepted and incorporated | - | 397-410 |
| 9 | There are some typo errors in the whole manuscript. Authors need to check and revise those mistakes. | Accepted and incorporated taking the editing services | - | - |

We look forward to hear from you regarding our resubmission. We would be glad to respond to any further questions and comments that you may have.

Sincerely,

Laxmi Kant Sharma

Central University of Rajasthan, NH-8, Bandar Sindri

Kishangarh, Ajmer, Rajasthan

laxmikant_evs@curaj.ac.in
